# Acknowledgment to Reviewers of *Medicines* in 2021

**DOI:** 10.3390/medicines9020012

**Published:** 2022-02-02

**Authors:** Medicines Editorial Office

**Affiliations:** MDPI AG, St. Alban-Anlage 66, 4052 Basel, Switzerland

Rigorous peer-reviews are the basis of high-quality academic publishing. Thanks to the great efforts of our reviewers, *Medicines* was able to maintain its standards for the high quality of its published papers. Thanks to the contribution of our reviewers, in 2021, the median time to first decision was 19 days and the median time to publication was 43.5 days. The editors would like to extend their gratitude and recognition to the following reviewers for their precious time and dedication, regardless of whether the papers they reviewed were finally published:
Aghajanyan, AnushNamgoong, SikAlmendra-Pegueros, RafaelNicolas, NabilAntipova, Veronica AlexandraNowak, AnnaBelluzzi, ElisaOlchawa, Magdalena M.Bhandarkar, Nikhil SureshPan, YijunBieńkowski, CarloPaulraj, Raja SinghBirgegård, GunnarPeregud-Pogorzelska, MałgorzataBlesson, ChellakkanPeriasamy, RameshBody-Malapel, MathildePetre, GabrielBóta, AndrásPetriczko, JanBruzzese, DarioPizarro-Sánchez, SoledadBurugupalli, SatvikaPloch-Jankowska, AnnaCaggiati, AlbertoPodlasek, RobertCalina, DanielaPrior, SarahCampa, Carlo CosimoProdromos, Chadwick C.Campiche, RemoQazi, Izhar HyderCampioni, PaoloQuante, MichaelChen, Mu-KuanQuintas, AlexandreCherif, El KhalilRadzki, DominikCho, Jang-HeeRaheem, Omer A.Concheiro-Moscoso, PatriciaRazvan-Cosmin, PetcaCormio, GennaroRiccioni, Maria ElenaCostantini, ElisabettaRodríguez González, IslayD′Andrea, SettimioRodríguez Gutiérrez, José LuisDariš, BarbaraRossiter, JackieDe Sanctis, Juan BautistaRyu, Jung SuDziubek-Rogowska, WiolettaSalinas-Martínez, Ana MaríaFleitas, TaniaSankararaman, SenthilkumarGallorini, MarialuciaSasaki, TakayaGómez-Mayordomo, VíctorSasso, Ferdinando CarloHao, LihongŠešelja Perišin, AnaHao, LiuyiShelat, Vishalkumar G.Hassan, AhmedShinomiya, NariyoshiHolder, AlvinSinger, Bernhard B.Iba, ToshiakiSoltész, BeátaIerardi, EnzoStambolija, VasilijeImam, SyedSteardo, LucaImamura, RyuSugimoto, MitsushigeJaramillo Ortiz, José ManuelSun, WeilunKaiafa, GeorgiaSurendran, Athira N.Kálai, TamasSwami Vetha, Berwin SinghKolota, AleksandraTabasum, SabaKotsapas, MichailTjioe, MarcoKutwin, MartaTripathi, TakshashilaL′homme, LaurentTseng, Kuang-WenLaffin, MichaelValeriani, FedericaLawal, MonsuratVassanelli, AuroraLayek, BuddhadevWaiker, PrashantLee, JeongminWang, FeilongLee, Sa RaWang, ShaobinLee, Yu-ChiWeekes, BrendanLi, XinboWollebo, Hassen S.Locatelli, GiuseppeWu, Chang-JerMangone, LuciaYang, ChunMarchesi, FedericoYano, YoshihikoMartins, DianaYu, AlanMay, AaronYu, QiuliyangMelendez, Johan H.Yu, YanboMenè, PaoloZechariah, SunithaMeštrović, TomislavZhao, JingyiMitchell, Cassie S.Zheng, XiaomingMlynarska, AgnieszkaZhu, ShengMotamed, CyrusZylicz, Zbigniew

